# Bis[μ-*O*-isopropyl (4-eth­oxy­phen­yl)dithio­phospho­nato-κ^2^ *S*:*S*′]bis­{[*O*-iso­propyl (4-eth­oxy­phen­yl)dithio­phos­phonato-κ^2^ *S*,*S*′]mercury(II)}

**DOI:** 10.1107/S1600536812046624

**Published:** 2012-11-17

**Authors:** Shirveen Sewpersad, Werner E. Van Zyl

**Affiliations:** a School of Chemistry and Physics, University of KwaZulu-Natal, Westville Campus, Private Bag X54001, Durban 4000, South Africa

## Abstract

The title compound, [Hg_2_(C_11_H_16_O_2_PS_2_)_4_], is a dinuclear complex with a distorted tetra­hedral geometry around each Hg^II^ atom. Although the two Hg^II^ atoms are surrounded by the same ligand, two different coordination modes are observed: one is chelating and the other bridging. The Hg—S bonds form two distinct pairs of long and short bonds. One pair includes both chelating and bridging Hg—S bonds with approximately equal bond lengths of 2.4042 (8) and 2.3997 (7) Å, respectively. The other pair is significantly longer at 2.9361 (9) and 2.8105 (8) Å, respectively. This pattern forms a center of inversion through the mol­ecule with an equal and opposite effect occurring at the other Hg^II^ atom. The S—Hg—S angles vary widely from 76.26 (2) to 154.65 (3)°, indicative of a distorted tetra­hedral arrangement of the S atoms around the Hg^II^ atom. The P—S bond lengths are 1.9681 (10) and 2.0519 (11)°, clearly indicating partial double-bond character in the former. The mol­ecule contains an inversion center situated between the two Hg^II^ atoms.

## Related literature

For information on dithio­phospho­nate compounds, see: Van Zyl & Fackler (2000 ▶); Van Zyl (2010 ▶). For examples of mercury(II) dithio­phospho­nate complexes, see: Gray *et al.* (2004*a* ▶,*b* ▶); Devillanova *et al.* (2006 ▶).
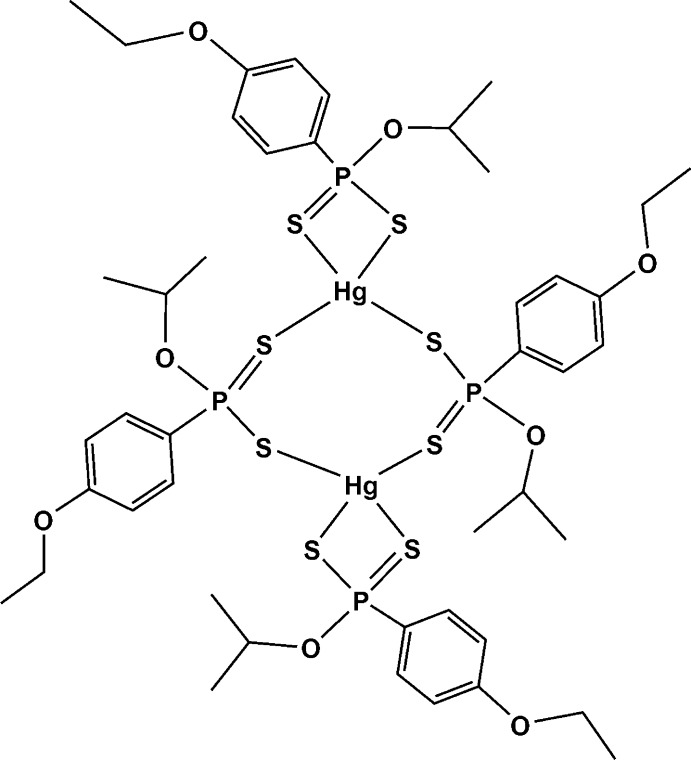


## Experimental

### 

#### Crystal data

[Hg_2_(C_11_H_16_O_2_PS_2_)_4_]
*M* *_r_* = 1502.49
Triclinic, 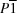

*a* = 11.079 (3) Å
*b* = 11.985 (3) Å
*c* = 12.253 (3) Å
α = 62.908 (4)°
β = 84.418 (4)°
γ = 80.862 (4)°
*V* = 1429.5 (6) Å^3^
*Z* = 1
Mo *K*α radiation
μ = 5.81 mm^−1^
*T* = 173 K
0.15 × 0.15 × 0.12 mm

#### Data collection

Bruker Kappa DUO APEXII diffractometer
Absorption correction: multi-scan (*SADABS*; Sheldrick, 1997 ▶) *T*_min_ = 0.476, *T*_max_ = 0.542
34157 measured reflections
6382 independent reflections
5622 reflections with *I* > 2σ(*I*)
*R*_int_ = 0.077

#### Refinement

*R*[*F* ^2^ > 2σ(*F* ^2^)] = 0.023
*wR*(*F* ^2^) = 0.049
*S* = 0.97
6382 reflections
304 parameters
H-atom parameters constrained
Δρ_max_ = 0.87 e Å^−3^
Δρ_min_ = −1.02 e Å^−3^

### 

Data collection: *APEX2* (Bruker, 2006 ▶); cell refinement: *SAINT* (Bruker, 2006 ▶); data reduction: *SAINT*; program(s) used to solve structure: *SHELXS97* (Sheldrick, 2008 ▶); program(s) used to refine structure: *SHELXL97* (Sheldrick, 2008 ▶); molecular graphics: *SHELXTL* (Sheldrick, 2008 ▶); software used to prepare material for publication: *SHELXL97*.

## Supplementary Material

Crystal structure: contains datablock(s) I, global. DOI: 10.1107/S1600536812046624/ff2088sup1.cif

Structure factors: contains datablock(s) I. DOI: 10.1107/S1600536812046624/ff2088Isup2.hkl

Additional supplementary materials:  crystallographic information; 3D view; checkCIF report

## Figures and Tables

**Fig. 1.**
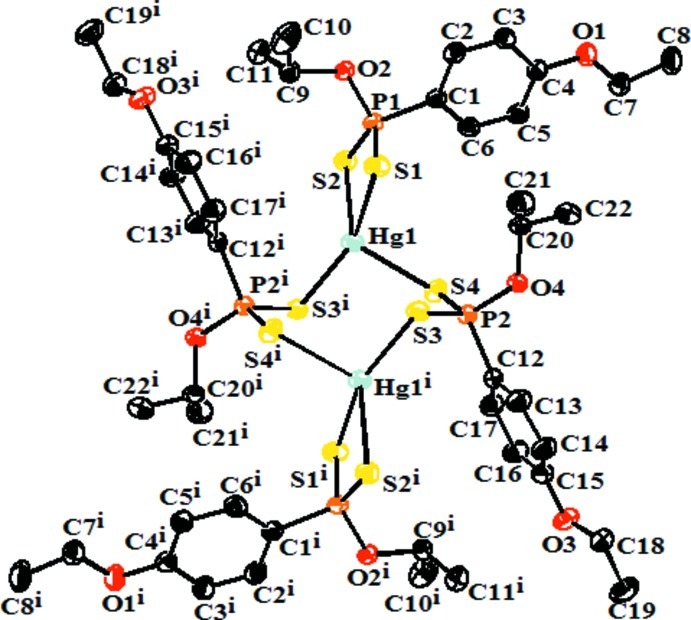
The ORTEP molecular structure of the title complex, shown with 50% probability.

## supplementary crystallographic information

### Comment

The phosphor-1,1,-dithiolate class of compounds is the heavier and softer congener of the more popular phosphonate derivatives. It contains the S_2_P functionality as a common feature and several sub-categories are known which include the dithiophosphato [S_2_P(OR')_2_]¯, (*R*' = typically alkyl), dithiophosphinato [S_2_P*R*_2_]¯ (*R* = alkyl or aryl), and dithiophosphonato [S_2_PR(OR')]¯, (*R* = typically aryl or ferrocenyl, *R*' = alkyl) monoanionic ligands. The latter may be described as a hybrid of the former two, and are also much less developed.

All known Hg(II) complexes with this ligand type are structurally more or less similar, as shown in the Scheme. The complexes are all dinuclear, mercuric, neutral, and 4-coordinate around each metal atom.

General and convenient methods to prepare dithiophosphonate salt derivatives have been reported (Van Zyl & Fackler, 2000). The title complex was formed through the reaction between two Hg^2+^ cations and four [S_2_P(4—C_6_H_4_OEt)(O^i^Pr)]¯ ligands, the formed complex feature an 8-membered Hg_2_P_2_S_4_ metallo-ring. Two of the ligands bind in a chelating manner and two ligands bind in a bridging manner, both types are anisobidentate, however, due to short and long pairings of the P—S bond and especially the Hg—S bonds.

### Experimental

A colorless methanol (40 ml) solution of NH_4_[S_2_P(O^i^Pr)(4-C_6_H_4_OEt)] (1.322 g, 4.506 mmol) was prepared. A second colorless solution of Hg(NO_3_)_2_.H_2_0 (0.772 g, 2.253 mmol) in deionized water (20 ml) was prepared, and added to the ligand solution with stirring over a period of 5 min. This resulted in a white precipitate indicating the formation of the title complex. The precipitate was collected by vacuum filtration, washed with water (3 *x* 10 ml) to remove NH_4_NO_3_ and allowed to dry under vacuum for a period of 3 hrs, yielding a dry, free-flowing white powder. Colourless crystals suitable for X-ray analysis were grown by the slow diffusion of hexane into a dichloromethane solution of the title complex. Yield: 76%. *M*.p. 117–118°C.

^31^P NMR (CDCl_3_): δ (p.p.m.): 100.73. ^1^H NMR (CDCl_3_): δ (p.p.m.): 7.93 (2*H*, dd, J(^31^P-^1^H) = 14.46 Hz, J(^1^H -^1^H) = 8.82 Hz, *o*-ArH), 6.91 (2*H*, dd, J(^31^P-^1^H) = 8.84 Hz, J(^1^H -^1^H) = 3.40 Hz, *m*-ArH), 5.23 (1*H*, d quart, J(^31^P-^1^H) = 20.82 Hz, J(^1^H -^1^H) = 6.19 Hz, CH), 4.05 (2*H*, quart, J(^1^H -^1^H) = 6.96 Hz, ArOCH_2_), 1.42 (6*H*, d, J(^1^H -^1^H) = 6.2 Hz, CH_3_), 1.39 (3*H*, t, J(^1^H -^1^H) = 6.96 Hz, ArOCH_2_CH_3_). ^13^C NMR (CDCl_3_): δ (p.p.m.): 162.27 (*p*-ArC), 132.15 (*m*-ArC), 130.40 (Ar—C_1_), 114.32 (*o*-ArC), 72.22 (CH), 64.03 (ArOCH_2_), 24.27 (CH_3_), 14.91 (ArOCH_2_CH_3_).

### Refinement

All hydrogen atoms were placed in idealized positions and refined with geometrical constraints and *U*_eq_ of 1.20–1.50 of parent C atom.. The structure was refined to *R* factor of 0.0227.

### Crystal data

**Table:**

[Hg_2_(C_11_H_16_O_2_PS_2_)_4_]	*Z* = 1
*M**_r_* = 1502.49	*F*(000) = 740
Triclinic, *P*1	*D*_x_ = 1.745 Mg m^−^^3^
Hall symbol: -P 1	Mo *K*α radiation, λ = 0.71073 Å
*a* = 11.079 (3) Å	Cell parameters from 34157 reflections
*b* = 11.985 (3) Å	θ = 1.9–27.4°
*c* = 12.253 (3) Å	µ = 5.81 mm^−^^1^
α = 62.908 (4)°	*T* = 173 K
β = 84.418 (4)°	Block, colourless
γ = 80.862 (4)°	0.15 × 0.15 × 0.12 mm
*V* = 1429.5 (6) Å^3^	

### Data collection

**Table:**

Bruker Kappa DUO APEXII diffractometer	6382 independent reflections
Radiation source: fine-focus sealed tube	5622 reflections with *I* > 2σ(*I*)
Graphite monochromator	*R*_int_ = 0.077
0.5° φ scans and ω scans	θ_max_ = 27.4°, θ_min_ = 1.9°
Absorption correction: multi-scan (*SADABS*; Sheldrick, 1997)	*h* = −14→14
*T*_min_ = 0.476, *T*_max_ = 0.542	*k* = −15→15
34157 measured reflections	*l* = −15→15

### Refinement

**Table:**

Refinement on *F*^2^	Primary atom site location: structure-invariant direct methods
Least-squares matrix: full	Secondary atom site location: difference Fourier map
*R*[*F*^2^ > 2σ(*F*^2^)] = 0.023	Hydrogen site location: inferred from neighbouring sites
*wR*(*F*^2^) = 0.049	H-atom parameters constrained
*S* = 0.97	*w* = 1/[σ^2^(*F*_o_^2^) + (0.017*P*)^2^] where *P* = (*F*_o_^2^ + 2*F*_c_^2^)/3
6382 reflections	(Δ/σ)_max_ = 0.002
304 parameters	Δρ_max_ = 0.87 e Å^−^^3^
0 restraints	Δρ_min_ = −1.02 e Å^−^^3^

### Special details

**Table:**

Geometry. All e.s.d.'s (except the e.s.d. in the dihedral angle between two l.s. planes) are estimated using the full covariance matrix. The cell e.s.d.'s are taken into account individually in the estimation of e.s.d.'s in distances, angles and torsion angles; correlations between e.s.d.'s in cell parameters are only used when they are defined by crystal symmetry. An approximate (isotropic) treatment of cell e.s.d.'s is used for estimating e.s.d.'s involving l.s. planes.
Refinement. Refinement of *F*^2^ against ALL reflections. The weighted *R*-factor *wR* and goodness of fit *S* are based on *F*^2^, conventional *R*-factors *R* are based on *F*, with *F* set to zero for negative *F*^2^. The threshold expression of *F*^2^ > σ(*F*^2^) is used only for calculating *R*-factors(gt) *etc*. and is not relevant to the choice of reflections for refinement. *R*-factors based on *F*^2^ are statistically about twice as large as those based on *F*, and *R*- factors based on ALL data will be even larger.

### Fractional atomic coordinates and isotropic or equivalent isotropic displacement parameters (Å^2^)

**Table:**

	*x*	*y*	*z*	*U*_iso_*/*U*_eq_	
Hg1	0.406211 (9)	0.860930 (11)	0.068281 (10)	0.03450 (5)	
S1	0.21870 (6)	0.87000 (7)	−0.02079 (7)	0.03184 (16)	
S2	0.33676 (6)	0.60580 (7)	0.21177 (6)	0.02963 (15)	
S3	0.47011 (6)	1.09499 (7)	−0.19071 (6)	0.02882 (15)	
S4	0.61332 (6)	0.79500 (7)	−0.05226 (6)	0.03264 (16)	
P1	0.21046 (6)	0.68074 (7)	0.08684 (6)	0.02416 (15)	
P2	0.59057 (6)	0.95292 (7)	−0.20573 (6)	0.02505 (15)	
O1	0.24135 (16)	0.4936 (2)	−0.28729 (18)	0.0369 (5)	
O2	0.07486 (14)	0.66471 (17)	0.13934 (16)	0.0283 (4)	
O3	1.05424 (16)	1.14120 (19)	−0.45478 (18)	0.0370 (5)	
O4	0.54046 (15)	0.93611 (17)	−0.31346 (16)	0.0286 (4)	
C1	0.2184 (2)	0.6092 (2)	−0.0143 (2)	0.0243 (6)	
C2	0.1144 (2)	0.6060 (3)	−0.0679 (2)	0.0339 (7)	
H2	0.0358	0.6332	−0.0434	0.041*	
C3	0.1250 (2)	0.5637 (3)	−0.1559 (3)	0.0366 (7)	
H3	0.0537	0.5607	−0.1911	0.044*	
C4	0.2396 (2)	0.5255 (3)	−0.1936 (2)	0.0280 (6)	
C5	0.3435 (2)	0.5230 (3)	−0.1371 (3)	0.0284 (6)	
H5	0.4219	0.4926	−0.1590	0.034*	
C6	0.3305 (2)	0.5655 (3)	−0.0487 (3)	0.0293 (6)	
H6	0.4015	0.5645	−0.0104	0.035*	
C7	0.3573 (2)	0.4493 (3)	−0.3265 (3)	0.0349 (7)	
H7A	0.3947	0.3713	−0.2581	0.042*	
H7B	0.4134	0.5141	−0.3530	0.042*	
C8	0.3361 (3)	0.4232 (4)	−0.4312 (3)	0.0503 (9)	
H8A	0.2828	0.3568	−0.4033	0.075*	
H8B	0.4146	0.3952	−0.4611	0.075*	
H8C	0.2972	0.5004	−0.4975	0.075*	
C9	0.0212 (2)	0.7226 (3)	0.2196 (3)	0.0328 (7)	
H9	0.0767	0.7807	0.2197	0.039*	
C10	−0.0988 (3)	0.7978 (3)	0.1647 (3)	0.0507 (9)	
H10A	−0.0837	0.8647	0.0824	0.076*	
H10B	−0.1397	0.8359	0.2167	0.076*	
H10C	−0.1511	0.7419	0.1595	0.076*	
C11	0.0099 (3)	0.6180 (3)	0.3468 (3)	0.0403 (7)	
H11A	−0.0408	0.5584	0.3459	0.060*	
H11B	−0.0283	0.6534	0.4017	0.060*	
H11C	0.0913	0.5737	0.3758	0.060*	
C12	0.7306 (2)	1.0142 (3)	−0.2752 (2)	0.0267 (6)	
C13	0.7268 (2)	1.1228 (3)	−0.3845 (3)	0.0338 (7)	
H13	0.6497	1.1674	−0.4171	0.041*	
C14	0.8323 (2)	1.1688 (3)	−0.4480 (3)	0.0343 (7)	
H14	0.8275	1.2432	−0.5240	0.041*	
C15	0.9455 (2)	1.1049 (3)	−0.3994 (3)	0.0307 (6)	
C16	0.9506 (2)	0.9983 (3)	−0.2874 (3)	0.0347 (7)	
H16	1.0276	0.9565	−0.2525	0.042*	
C17	0.8446 (2)	0.9522 (3)	−0.2259 (3)	0.0331 (6)	
H17	0.8493	0.8780	−0.1498	0.040*	
C18	1.0515 (3)	1.2395 (3)	−0.5783 (3)	0.0378 (7)	
H18A	1.0037	1.3180	−0.5815	0.045*	
H18B	1.0125	1.2142	−0.6316	0.045*	
C19	1.1816 (3)	1.2612 (3)	−0.6217 (3)	0.0503 (9)	
H19A	1.2200	1.2835	−0.5669	0.075*	
H19B	1.1821	1.3303	−0.7052	0.075*	
H19C	1.2271	1.1839	−0.6213	0.075*	
C20	0.4382 (2)	0.8642 (3)	−0.2971 (3)	0.0314 (7)	
H20	0.4191	0.8146	−0.2077	0.038*	
C21	0.3287 (3)	0.9560 (3)	−0.3587 (3)	0.0468 (8)	
H21A	0.3069	1.0137	−0.3213	0.070*	
H21B	0.2598	0.9097	−0.3487	0.070*	
H21C	0.3479	1.0047	−0.4462	0.070*	
C22	0.4828 (3)	0.7755 (3)	−0.3523 (3)	0.0415 (8)	
H22A	0.5113	0.8238	−0.4371	0.062*	
H22B	0.4159	0.7305	−0.3512	0.062*	
H22C	0.5504	0.7144	−0.3045	0.062*	

### Atomic displacement parameters (Å^2^)

**Table:**

	*U*^11^	*U*^22^	*U*^33^	*U*^12^	*U*^13^	*U*^23^
Hg1	0.03746 (7)	0.03923 (8)	0.03315 (8)	−0.02021 (5)	0.00380 (5)	−0.01779 (6)
S1	0.0325 (4)	0.0254 (4)	0.0331 (4)	−0.0065 (3)	−0.0045 (3)	−0.0077 (3)
S2	0.0288 (3)	0.0303 (4)	0.0288 (4)	−0.0053 (3)	−0.0040 (3)	−0.0112 (3)
S3	0.0272 (3)	0.0276 (4)	0.0345 (4)	−0.0068 (3)	0.0003 (3)	−0.0155 (3)
S4	0.0339 (4)	0.0301 (4)	0.0283 (4)	−0.0051 (3)	0.0038 (3)	−0.0090 (3)
P1	0.0220 (3)	0.0250 (4)	0.0266 (4)	−0.0065 (3)	0.0011 (3)	−0.0119 (3)
P2	0.0260 (3)	0.0271 (4)	0.0240 (4)	−0.0085 (3)	0.0018 (3)	−0.0120 (3)
O1	0.0292 (10)	0.0557 (15)	0.0360 (12)	−0.0048 (9)	0.0007 (8)	−0.0298 (11)
O2	0.0236 (9)	0.0348 (12)	0.0328 (11)	−0.0098 (8)	0.0067 (7)	−0.0200 (9)
O3	0.0271 (10)	0.0425 (13)	0.0369 (12)	−0.0111 (9)	0.0067 (8)	−0.0133 (10)
O4	0.0341 (10)	0.0324 (12)	0.0245 (10)	−0.0160 (8)	0.0035 (8)	−0.0142 (9)
C1	0.0239 (12)	0.0243 (15)	0.0235 (14)	−0.0060 (10)	−0.0003 (10)	−0.0089 (12)
C2	0.0224 (13)	0.0484 (19)	0.0350 (17)	−0.0050 (12)	0.0019 (11)	−0.0225 (15)
C3	0.0250 (14)	0.059 (2)	0.0359 (17)	−0.0084 (13)	−0.0008 (11)	−0.0288 (16)
C4	0.0303 (14)	0.0290 (16)	0.0255 (15)	−0.0074 (12)	0.0009 (11)	−0.0120 (13)
C5	0.0237 (13)	0.0296 (16)	0.0339 (16)	−0.0023 (11)	0.0011 (11)	−0.0167 (13)
C6	0.0230 (13)	0.0343 (17)	0.0349 (16)	−0.0031 (11)	−0.0036 (11)	−0.0189 (14)
C7	0.0312 (14)	0.0427 (19)	0.0370 (17)	−0.0051 (13)	0.0023 (12)	−0.0236 (15)
C8	0.0424 (18)	0.078 (3)	0.043 (2)	−0.0038 (17)	0.0040 (14)	−0.041 (2)
C9	0.0346 (15)	0.0348 (18)	0.0380 (17)	−0.0109 (13)	0.0089 (12)	−0.0238 (15)
C10	0.0457 (19)	0.038 (2)	0.053 (2)	0.0070 (15)	0.0093 (16)	−0.0137 (17)
C11	0.0387 (16)	0.050 (2)	0.0367 (18)	−0.0115 (14)	0.0065 (13)	−0.0233 (16)
C12	0.0282 (13)	0.0327 (17)	0.0240 (14)	−0.0092 (11)	0.0026 (11)	−0.0158 (13)
C13	0.0278 (14)	0.0386 (18)	0.0292 (16)	−0.0061 (12)	−0.0039 (11)	−0.0089 (14)
C14	0.0317 (14)	0.0370 (18)	0.0263 (16)	−0.0104 (12)	0.0000 (11)	−0.0055 (14)
C15	0.0270 (14)	0.0344 (17)	0.0348 (17)	−0.0095 (12)	0.0032 (11)	−0.0182 (14)
C16	0.0245 (14)	0.0361 (18)	0.0390 (18)	−0.0012 (12)	−0.0015 (12)	−0.0135 (15)
C17	0.0348 (15)	0.0306 (17)	0.0285 (16)	−0.0057 (12)	−0.0008 (12)	−0.0081 (13)
C18	0.0398 (16)	0.0410 (19)	0.0348 (17)	−0.0145 (14)	0.0088 (13)	−0.0179 (15)
C19	0.0424 (18)	0.056 (2)	0.049 (2)	−0.0184 (16)	0.0165 (15)	−0.0196 (18)
C20	0.0314 (14)	0.0397 (18)	0.0285 (16)	−0.0188 (13)	0.0034 (11)	−0.0162 (14)
C21	0.0342 (16)	0.061 (2)	0.054 (2)	−0.0060 (15)	−0.0022 (14)	−0.0326 (19)
C22	0.0429 (17)	0.043 (2)	0.048 (2)	−0.0182 (15)	0.0028 (14)	−0.0249 (17)

### Geometric parameters (Å, º)

**Table:**

Hg1—S3^i^	2.3997 (7)	C9—C11	1.499 (4)
Hg1—S1	2.4042 (8)	C9—C10	1.509 (4)
Hg1—S4	2.8105 (8)	C9—H9	1.0000
Hg1—S2	2.9361 (9)	C10—H10A	0.9800
S1—P1	2.0519 (11)	C10—H10B	0.9800
S2—P1	1.9681 (10)	C10—H10C	0.9800
S3—P2	2.0568 (10)	C11—H11A	0.9800
S3—Hg1^i^	2.3998 (7)	C11—H11B	0.9800
S4—P2	1.9699 (11)	C11—H11C	0.9800
P1—O2	1.5807 (17)	C12—C13	1.377 (4)
P1—C1	1.788 (3)	C12—C17	1.398 (4)
P2—O4	1.5848 (18)	C13—C14	1.382 (4)
P2—C12	1.791 (2)	C13—H13	0.9500
O1—C4	1.363 (3)	C14—C15	1.393 (4)
O1—C7	1.435 (3)	C14—H14	0.9500
O2—C9	1.476 (3)	C15—C16	1.383 (4)
O3—C15	1.357 (3)	C16—C17	1.380 (4)
O3—C18	1.433 (3)	C16—H16	0.9500
O4—C20	1.475 (3)	C17—H17	0.9500
C1—C6	1.375 (3)	C18—C19	1.507 (4)
C1—C2	1.395 (3)	C18—H18A	0.9900
C2—C3	1.374 (4)	C18—H18B	0.9900
C2—H2	0.9500	C19—H19A	0.9800
C3—C4	1.389 (4)	C19—H19B	0.9800
C3—H3	0.9500	C19—H19C	0.9800
C4—C5	1.389 (3)	C20—C21	1.500 (4)
C5—C6	1.379 (4)	C20—C22	1.501 (4)
C5—H5	0.9500	C20—H20	1.0000
C6—H6	0.9500	C21—H21A	0.9800
C7—C8	1.498 (4)	C21—H21B	0.9800
C7—H7A	0.9900	C21—H21C	0.9800
C7—H7B	0.9900	C22—H22A	0.9800
C8—H8A	0.9800	C22—H22B	0.9800
C8—H8B	0.9800	C22—H22C	0.9800
C8—H8C	0.9800		
			
S3^i^—Hg1—S1	154.65 (3)	C10—C9—H9	109.5
S3^i^—Hg1—S4	91.92 (3)	C9—C10—H10A	109.5
S1—Hg1—S4	112.32 (3)	C9—C10—H10B	109.5
S3^i^—Hg1—S2	109.11 (2)	H10A—C10—H10B	109.5
S1—Hg1—S2	76.26 (2)	C9—C10—H10C	109.5
S4—Hg1—S2	97.22 (2)	H10A—C10—H10C	109.5
P1—S1—Hg1	92.17 (3)	H10B—C10—H10C	109.5
P1—S2—Hg1	79.38 (3)	C9—C11—H11A	109.5
P2—S3—Hg1^i^	97.48 (3)	C9—C11—H11B	109.5
P2—S4—Hg1	94.61 (4)	H11A—C11—H11B	109.5
O2—P1—C1	100.56 (10)	C9—C11—H11C	109.5
O2—P1—S2	114.83 (8)	H11A—C11—H11C	109.5
C1—P1—S2	114.39 (9)	H11B—C11—H11C	109.5
O2—P1—S1	107.71 (8)	C13—C12—C17	118.5 (2)
C1—P1—S1	106.96 (9)	C13—C12—P2	119.0 (2)
S2—P1—S1	111.53 (4)	C17—C12—P2	122.4 (2)
O4—P2—C12	100.50 (10)	C12—C13—C14	121.8 (3)
O4—P2—S4	113.62 (8)	C12—C13—H13	119.1
C12—P2—S4	113.57 (10)	C14—C13—H13	119.1
O4—P2—S3	104.78 (8)	C13—C14—C15	119.2 (3)
C12—P2—S3	108.57 (10)	C13—C14—H14	120.4
S4—P2—S3	114.56 (4)	C15—C14—H14	120.4
C4—O1—C7	117.9 (2)	O3—C15—C16	116.5 (2)
C9—O2—P1	120.41 (15)	O3—C15—C14	124.0 (3)
C15—O3—C18	117.1 (2)	C16—C15—C14	119.6 (2)
C20—O4—P2	123.35 (15)	C17—C16—C15	120.6 (3)
C6—C1—C2	118.4 (2)	C17—C16—H16	119.7
C6—C1—P1	119.55 (19)	C15—C16—H16	119.7
C2—C1—P1	121.8 (2)	C16—C17—C12	120.3 (3)
C3—C2—C1	120.4 (2)	C16—C17—H17	119.9
C3—C2—H2	119.8	C12—C17—H17	119.9
C1—C2—H2	119.8	O3—C18—C19	107.8 (2)
C2—C3—C4	120.3 (2)	O3—C18—H18A	110.1
C2—C3—H3	119.8	C19—C18—H18A	110.1
C4—C3—H3	119.8	O3—C18—H18B	110.1
O1—C4—C5	124.2 (2)	C19—C18—H18B	110.1
O1—C4—C3	116.0 (2)	H18A—C18—H18B	108.5
C5—C4—C3	119.8 (2)	C18—C19—H19A	109.5
C6—C5—C4	118.7 (2)	C18—C19—H19B	109.5
C6—C5—H5	120.6	H19A—C19—H19B	109.5
C4—C5—H5	120.6	C18—C19—H19C	109.5
C1—C6—C5	122.2 (2)	H19A—C19—H19C	109.5
C1—C6—H6	118.9	H19B—C19—H19C	109.5
C5—C6—H6	118.9	O4—C20—C21	108.4 (2)
O1—C7—C8	108.0 (2)	O4—C20—C22	105.6 (2)
O1—C7—H7A	110.1	C21—C20—C22	113.5 (3)
C8—C7—H7A	110.1	O4—C20—H20	109.7
O1—C7—H7B	110.1	C21—C20—H20	109.7
C8—C7—H7B	110.1	C22—C20—H20	109.7
H7A—C7—H7B	108.4	C20—C21—H21A	109.5
C7—C8—H8A	109.5	C20—C21—H21B	109.5
C7—C8—H8B	109.5	H21A—C21—H21B	109.5
H8A—C8—H8B	109.5	C20—C21—H21C	109.5
C7—C8—H8C	109.5	H21A—C21—H21C	109.5
H8A—C8—H8C	109.5	H21B—C21—H21C	109.5
H8B—C8—H8C	109.5	C20—C22—H22A	109.5
O2—C9—C11	107.6 (2)	C20—C22—H22B	109.5
O2—C9—C10	106.3 (2)	H22A—C22—H22B	109.5
C11—C9—C10	114.4 (2)	C20—C22—H22C	109.5
O2—C9—H9	109.5	H22A—C22—H22C	109.5
C11—C9—H9	109.5	H22B—C22—H22C	109.5
			
S3^i^—Hg1—S1—P1	−110.80 (6)	C1—C2—C3—C4	−0.9 (5)
S4—Hg1—S1—P1	87.19 (4)	C7—O1—C4—C5	2.9 (4)
S2—Hg1—S1—P1	−5.07 (3)	C7—O1—C4—C3	−177.9 (3)
S3^i^—Hg1—S2—P1	159.53 (3)	C2—C3—C4—O1	−175.5 (3)
S1—Hg1—S2—P1	5.38 (3)	C2—C3—C4—C5	3.7 (4)
S4—Hg1—S2—P1	−105.91 (3)	O1—C4—C5—C6	175.7 (2)
S3^i^—Hg1—S4—P2	−104.88 (4)	C3—C4—C5—C6	−3.5 (4)
S1—Hg1—S4—P2	67.51 (4)	C2—C1—C6—C5	2.3 (4)
S2—Hg1—S4—P2	145.58 (3)	P1—C1—C6—C5	−172.3 (2)
Hg1—S2—P1—O2	−129.47 (8)	C4—C5—C6—C1	0.5 (4)
Hg1—S2—P1—C1	114.96 (9)	C4—O1—C7—C8	−178.8 (3)
Hg1—S2—P1—S1	−6.59 (4)	P1—O2—C9—C11	−108.9 (2)
Hg1—S1—P1—O2	134.79 (7)	P1—O2—C9—C10	128.1 (2)
Hg1—S1—P1—C1	−117.84 (8)	O4—P2—C12—C13	56.8 (2)
Hg1—S1—P1—S2	7.92 (5)	S4—P2—C12—C13	178.5 (2)
Hg1—S4—P2—O4	−105.45 (8)	S3—P2—C12—C13	−52.8 (2)
Hg1—S4—P2—C12	140.49 (9)	O4—P2—C12—C17	−119.7 (2)
Hg1—S4—P2—S3	14.94 (4)	S4—P2—C12—C17	2.0 (3)
Hg1^i^—S3—P2—O4	−165.11 (7)	S3—P2—C12—C17	130.7 (2)
Hg1^i^—S3—P2—C12	−58.41 (10)	C17—C12—C13—C14	2.3 (4)
Hg1^i^—S3—P2—S4	69.71 (4)	P2—C12—C13—C14	−174.3 (2)
C1—P1—O2—C9	−173.8 (2)	C12—C13—C14—C15	−1.1 (4)
S2—P1—O2—C9	62.9 (2)	C18—O3—C15—C16	171.0 (3)
S1—P1—O2—C9	−62.0 (2)	C18—O3—C15—C14	−9.7 (4)
C12—P2—O4—C20	167.3 (2)	C13—C14—C15—O3	179.3 (3)
S4—P2—O4—C20	45.6 (2)	C13—C14—C15—C16	−1.4 (4)
S3—P2—O4—C20	−80.2 (2)	O3—C15—C16—C17	−178.1 (2)
O2—P1—C1—C6	−158.7 (2)	C14—C15—C16—C17	2.5 (4)
S2—P1—C1—C6	−35.1 (2)	C15—C16—C17—C12	−1.2 (4)
S1—P1—C1—C6	89.0 (2)	C13—C12—C17—C16	−1.2 (4)
O2—P1—C1—C2	26.9 (2)	P2—C12—C17—C16	175.3 (2)
S2—P1—C1—C2	150.5 (2)	C15—O3—C18—C19	−178.4 (2)
S1—P1—C1—C2	−85.5 (2)	P2—O4—C20—C21	107.9 (2)
C6—C1—C2—C3	−2.1 (4)	P2—O4—C20—C22	−130.0 (2)
P1—C1—C2—C3	172.4 (2)		

Symmetry code: (i) −*x*+1, −*y*+2, −*z*.
